# Comparison of Sensory Profiling by Descriptive Analysis, Free-Choice Profiling, and Polarized Sensory Positioning on Bottled Water

**DOI:** 10.3390/foods12081579

**Published:** 2023-04-07

**Authors:** JeongAe Heo, Sang Sook Kim, Mi-Ran Kim, Han Sub Kwak

**Affiliations:** 1 Food Processing Research Group, Korea Food Research Institute, Wanju-gun 55365, Republic of Korea; heo.jeongae@kfri.re.kr (J.H.); sskim@kfri.re.kr (S.S.K.); 2 Department of Food Science and Nutrition, The Catholic University of Korea, Bucheon-si 14662, Republic of Korea; 3 KFRI School, University of Science and Technology, Wanju-gun 55365, Republic of Korea

**Keywords:** consumer-oriented profiling, free-choice profiling, polarized sensory positioning, descriptive analysis, water, mineral content

## Abstract

Consumer-oriented rapid profiling methodologies, including free-choice profiling (FCP) and polarized sensory positioning (PSP), have been studied in recent decades, highlighting alternative aspects of conventional descriptive analysis (DA). In the present study, water samples were evaluated using DA, FCP, and PSP with open-ended questions to compare the sensory profiles. Ten bottled water samples and one filtered water sample were evaluated by a trained panel for DA (n = 11), a semi-trained panel for FCP (n = 16), and naïve consumers for PSP (n = 63). The results were analyzed using principal component analysis for DA and multiple factor analysis for FCP and PSP data. The water samples were discriminated by their total mineral content, which was mainly associated with heavy mouthfeel. The overall discrimination patterns for the samples were similar between FCP and PSP, whereas DA showed different patterns. Sample discrimination through confidence ellipses from DA, FCP, and PSP showed that two consumer-oriented methodologies distinguished samples more clearly than DA. Throughout this study, consumer-oriented profiling methodologies were able to be used to investigate sensory profiles and provide rich information on consumer-derived sensory attributes even for subtly different samples.

## 1. Introduction

Various types of rapid sensory techniques have been used to assess the sensory profiles of food and products [1]. In particular, these various sensory techniques have been compared to obtain more insight about not only products but also sensory methodologies themselves [2,3,4]. In recent decades, various consumer-oriented rapid methodologies have been studied as alternatives to conventional sensory profiling methods [5,6]. Two of the main consumer-oriented methodologies are polarized sensory positioning (PSP) and free-choice profiling (FCP). The first, PSP, is a holistic method used to evaluate the relative similarity of samples compared to pre-selected reference points, called “poles” [7,8]. Teillet et al. [7] evaluated water samples, including 17 bottled water and 13 tap water samples, using the sensory profile, temporal dominance of sensation, sorting task, and PSP. The authors reported that PSP could discriminate water samples better than other methodologies because PSP results indicated a comparative relationship among samples using a holistic approach, especially in separating low and high mineralized water more than the sensory profile. A recent study by Moelich et al. [9] confirmed that the results of descriptive analysis (DA) were similar to the results of global PSP (RV coefficient of 0.95) and PSP for only aroma attributes (RV coefficient of 0.94) by a trained panel, even when a large set of herbal tea infusion samples was used. In addition, polarized projective mapping, which combines both PSP and projective mapping, was studied by Ares et al. [10], extending the applicable concept of PSP.

The other consumer-oriented methodology, FCP, allows consumers to generate sensory attributes individually without any consensus and evaluate samples based on their own attributes [11,12]. Each consumer uses different sensory attributes because of the nature of the evaluation. However, the consumer assessors were thought to perceive the samples in similar patterns, even if their expression of the sensory attributes varied, as suggested by Guàrdia et al. [13]. This implies that consumer-oriented profiling methods, such as FCP, enable researchers to gain insight into consumers’ perspectives on the samples. Several studies have been conducted to compare the results of FCP with DA [14,15] or check-all-that-apply [16] for various samples, to determine whether FCP can be alternatively used to profile the sensory attributes.

Although consumer-oriented methodologies have been studied as mentioned above, there are still few studies that compare sensory methodologies, preventing a comprehensive understanding of consumer-oriented methods. In this study, three methodologies with different panels were employed: DA, a classical sensory method used to identify and evaluate the sensory attributes of products with eight–twenty highly trained panelists [17]; FCP with semi-trained assessors; and PSP with naïve consumers participating in the evaluation. Both FCP and PSP approaches involve consumer-based aspects. FCP allows consumers to derive sensory attributes and evaluate the intensity of individual sensory characteristics, whereas PSP focuses on the overall similarity among samples rather than focusing on the specific sensory attributes. Therefore, this study aimed to compare sensory profiles and discriminate samples of the three methods by adding open-ended questions to PSP to elicit sensory attributes. Water samples were used for this research to confirm whether the results could be explained even in samples with subtle differences.

## 2. Materials and Methods

### 2.1. Sample and Sample Preparation

This study used 11 samples, comprising 10 commercial bottled water samples and 1 filtered water sample (Table 1). All samples, except for the filtered water sample (E1), were available in the local or online market. All commercial bottled water samples were stored at room temperature (22 °C). The filtered water sample (E1) was collected in a Duran glass bottle with a cap (1000 mL; DWK Life Sciences, Mainz, Germany) 1 day prior to each evaluation. Thirty grams of each sample was poured into a transparent plastic cup (30 mL; Newpack, Gyeonggido, Republic of Korea) 2 h prior to the evaluation. Each sample was labeled with a 3-digit random number to prevent bias.

### 2.2. Chemical Analysis of Samples

The mineral contents of Ca^2+^, Na^+^, K^+^, and Mg^2+^ in the water samples were analyzed using inductively coupled plasma optical emission spectroscopy (ICP-OES, Avio^®^ 500; PerkinElmer Inc., Waltham, MA, USA) at the Korea Food Research Institute (KFRI, Wanju-gun, Republic of Korea). The F^−^ content was quantified using ion chromatography (Thermo Scientific Dionex ICS-5000+; Thermo Fisher Scientific, Waltham, MA, USA) at the Korea Institute of Geoscience and Mineral Resources (Daejeon, Republic of Korea). The mineral compositions of the water samples are listed in Table 1. All the analyses were conducted according to the analysis of mineral contents from the Ministry of Food and Drug Safety, Republic of Korea [18].

### 2.3. Procedures

Each sample was provided every 5 min in sequential monadic order using Williams’ Latin-square design for the DA, FCP, and PSP [19]. The panelists were encouraged to taste half of the sample (~15 g) for each sip. The evaluations were conducted using a sensory data collection program (Compusense Inc., Guelph, ON, Canada) in an individual sensory booth at the KFRI. All participants provided informed consent and voluntarily agreed to participate in this study. They were able to withdraw from the test at any time without providing a reason. The experiment followed the guidelines of the Institutional Review Board of the KFRI.

#### 2.3.1. Descriptive Analysis

Twenty panelists who had previous experience in the DA participated in the screening test and conducted preliminary performance evaluations to test the ability to detect and distinguish between five basic tastes. Among them, 11 panelists (females, aged 40–50 years) who could meet our training schedule were selected for DA panelists. The panelists participated in six 2 h training sessions. During the training sessions, panelists generated sensory attributes and established definitions and reference materials for each attribute. The intensity of the reference material was calculated as the mean intensity of the panelists. Ten sensory attributes were developed, comprising two for appearance, five for taste/flavor, and three for mouthfeel (Table 2). The evaluations were conducted using a 15 cm line scale (0: none, 15: very strong). The final evaluation was conducted in quadruplicate after training.

#### 2.3.2. Free-Choice Profiling

Sixteen panelists (females = 12, aged 20 years) participated in the FCP evaluation. The panelists freely generated their own sensory attributes for the water samples. They were recommended not to use hedonic-related and comparative attributes. After generating the sensory attributes, a list of sensory attributes was individually provided to each panelist. Over the next four sessions, they tasted the samples again and added or removed attributes. The removal of synonyms and antonym attributes was also encouraged. The evaluations were conducted using a 15 cm line scale (0: none, 15: very strong) with panelist-derived anchor terms. The sample evaluation was repeated four times.

#### 2.3.3. Polarized Sensory Positioning

A total of 63 consumers (females = 37, aged 20–50 years) participated in the PSP evaluation. Prior to the evaluation, a brief orientation regarding the evaluation process was held for 10 min, and the evaluation was conducted once for 50 min. Poles A, B, and C were selected based on variations in mineral content (Ca^2+^ and Mg^2+^). Thus, the A1 (low; pole A), B2 (moderate; pole B), and D4 (high; pole C) samples were selected for the pole. Consumers evaluated the similarity of poles A, B, and C with each testing sample using a 15 cm line scale (0: same, 15: totally different). The sensory attributes, except for comparative terms or subjective terms, were provided in response to open-ended questions.

### 2.4. Statistical Analysis

Analysis of variance (ANOVA) was conducted on the DA results. Student–Newman–Keul’s multiple comparison test was applied to identify statistical significance across the samples (*p* < 0.05). Principal component analysis (PCA) was conducted to visually summarize the correlation between sensory attributes and water samples using the SensoMineR package. Multiple factor analysis (MFA) using the FactoMineR package was performed to analyze the data obtained from the FCP. The sensory attributes derived from each panelist were selected according to the correlation coefficients (>0.7) and analyzed to present the sample discrimination patterns of the product map referenced by Lee et al. [20]. MFA was also performed to investigate PSP data from different groups of variables (consumers). The terms from the open-ended questions generated during PSP were reviewed by two researchers and finalized by consensus. After this process, terms mentioned by more than 5% of consumers were selected and projected as supplementary data to understand sample configuration as per Symoneaux et al. [21]. Confidence ellipses were elicited to examine the discrimination among samples in all product maps: PCA map of DA and MFA maps of FCP and PSP. Additionally, the RV coefficient was calculated using MFA to examine the similarity of the results obtained with the three methodologies (DA, FCP, and PSP). The RV coefficient has a value of 0 to 1; the closer it is to 1, the more similar the two configurations [22]. All analyses were conducted using R studio (ver. 3.6.3.; RStudio, Inc., Boston, MA, USA).

## 3. Results

### 3.1. DA Results

**Figure 1:**
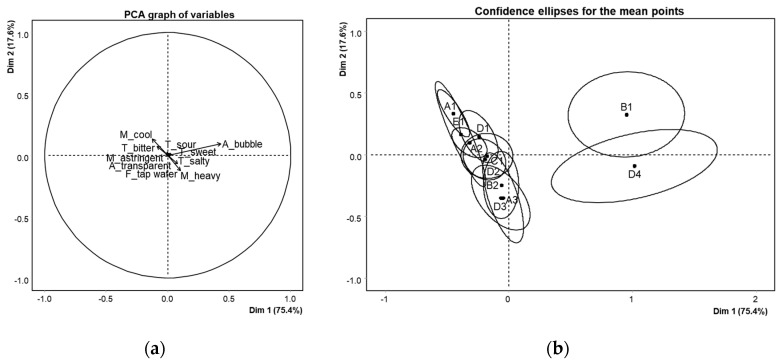
(**a**) Principal component analysis (PCA) of descriptive analysis (DA); (**b**) confidence ellipses of sample configurations of DA.

The ANOVA (Table 3) revealed significant differences in bubbles, salty taste, and heavy feeling among the 10 attributes (*p* < 0.05). The B1 and D4 samples had higher bubble attributes, whereas the D4 sample had higher salty taste and heavy feeling attributes. This result was thought to be affected by the mineral content of D4 samples, which was the highest among the samples (Table 1). The PCA result (Figure 1) explained a total of 93.0% of the variance, with PC1 (75.4%) and PC2 (17.6%) accounting for the majority of the variance. Overall, the positive axis of PC1 was mainly explained by bubbles, salty taste, and heavy feeling. Samples B1 and D4 were positioned on the positive axis of PC1, whereas the other samples (n = 9) were positioned on the opposite side. D4 was closely related to the salty taste and heavy feeling, which was influenced by the highest amount of total minerals induced by the highest amounts of Ca^2+^ and Mg^2+^ in D4. The B1 sample was associated with the bubble attribute, and this fact seemed to be due to the type of water in the samples (deep sea drinking water) rather than the amount of minerals. In contrast, the confidence ellipses of samples on the negative axis of PC1 (A1, A2, A3, B2, C1, D1, D2, D3, and E1) overlapped with each other, indicating that there were no significant differences in attributes among them.

### 3.2. FCP Results

**Figure 2:**
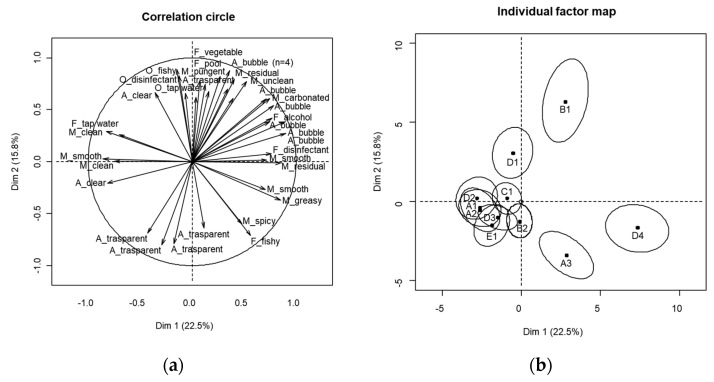
(**a**) Multiple factor analysis of free-choice profiling (FCP); (**b**) confidence ellipses of sample configurations of FCP.

A total of 159 sensory descriptors, including overlapping terms, were generated by 16 panelists, resulting in an average of approximately 10 attributes per panelist (range: 5–16; results not shown). Using MFA, 38.3% of the variance was explained, with 22.5% by Dim 1 and 15.8% by Dim 2 (Figure 2). The bubble attributes were primarily located on the positive axes of Dim 1 and Dim 2, correlating with sample B1. In contrast, samples A3 and D4 were positioned on Dim 1(+) and Dim 2(−), which were characterized by a disinfectant flavor, fishy flavor, and greasy mouthfeel. Although samples C1 and D2 were slightly positioned on the positive side of Dim 1, most samples (A1, A2, B2, C1, D2, D3, and E1) were located on the negative axis of Dim 1 and Dim 2 and overlapped. These samples were uncharacterized by other distinct sensory attributes, except for the transparent appearance. The confidence ellipses of the samples showed that A3, B1, D1, and D4 were clearly discriminated, whereas the other samples overlapped.

### 3.3. PSP Results

**Figure 3:**
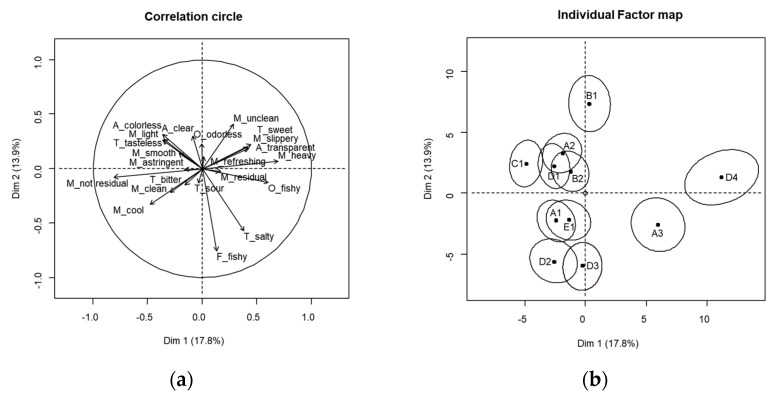
(**a**) Multiple factor analysis of polarized sensory positioning (PSP); (**b**) confidence ellipses of sample configurations of PSP.

Only terms generated by >5% of the consumers were used for the MFA. Thus, 22 terms were projected on the MFA map (Figure 3). MFA results showed that 31.7% of the variance was explained, with 17.8% explained by Dim 1 and 13.9% by Dim 2. Dim 1 was primarily characterized by mouthfeel, as both heavy and unclean mouthfeel were positioned on the positive axis, whereas clean mouthfeel and no residual mouthfeel were located on the negative axis. The negative axis of Dim 2 was mainly characterized by a salty taste and fishy flavor, correlating with samples A3 and D4. Sample B1, along with these samples, was distinguished from the others in the overall sample plot. Confidence ellipse results indicated that the remaining samples were roughly divided into two groups: A2/B2/C1/D1 and A1/D2/D3/E1. These samples had relatively bland characteristics, such as a colorless appearance, tastelessness, clean mouthfeel, and no residual mouthfeel.

### 3.4. Comparison of Three Methodologies

Overall, the samples were primarily differentiated by Dim 1, with samples B1 and D4 on the positive axis of Dim 1 and the other samples on the negative axis of Dim 1, regardless of the method used. The sample plots for each methodology also indicated that more samples were distinguished from each other in the FCP and PSP as a result of the confidence ellipses. For instance, sample A3 was not distinguished in DA, even though it contained the second-highest amount of total mineral content. In the DA plot, the purified water sample (C1) and filtered water sample (E1) were overlapped, whereas clear discrimination was found in the PSP. The RV coefficients derived from the MFA results determined the relationships between the listed methodologies in Table 4. The highest RV coefficients were in FCP and PSP (0.94), followed by DA and FCP (0.74), whereas there was moderate correlation between DA and PSP (0.65). The overall results of the sensory map for the three methodologies showed that the samples were more separated in FCP and PSP than in the DA.

## 4. Discussion

The sensory maps obtained from each methodology primarily differentiated samples based on their mineral content, rather than ingredients or manufacturing country, which is consistent with the results of previous water studies [7,23]. Not only the bubble attributes but also the higher mineral content of samples B1 and D4 influenced the overall sample configurations. Specifically, samples with large amounts of minerals (B1 and D4) were associated with a salty taste in the results of DA and PSP, similar to the results from Teillet et al. [7]. In terms of sensory attributes, both FCP and DA generated bubbles and had a transparent appearance, with the FCP generating various consumer-oriented terms not mentioned in DA (e.g., a disinfectant flavor, fish flavor, greasy mouthfeel, and residual mouthfeel). Similarly, fishy odor/flavor, slippery mouthfeel, non-residual mouthfeel, and unclean mouthfeel were also mentioned in open-ended questions in PSP. Generally, semi-trained panelists for FCP and consumer panelists for PSP provided sensory characteristics from their own language based on their personal traits and experiences with the evaluated products [24,25,26]. This heterogeneous nature of the consumers contributed to a more separated sample plot, which could not be achieved using only consensus attributes from DA.

The overall sample discrimination results might be also influenced by the different scale usage tendencies of each methodology. In DA, the established intensities of reference materials in sensory attributes could limit the scope of scale use, leading to less distinct differences in specific sensory attributes. Conversely, consumer assessors used their own sensory attributes and anchors at both extremes of the scale. Since there were no guidelines or training for scale use during FCP evaluations, consumers tended to use a wider range of scales. Similarly, the consumer assessors of PSP were likely to use a wider range of scales since they evaluated the samples based on perceived holistic similarity, rather than evaluating specific single attributes. Lawless et al. [27] reported that untrained subjects tended to use a broader range of scales even when there were small differences among the samples. Unlike DA, which requires objective intensity of each sensory attribute, the relativity of scale usage in FCP and PSP appeared to partly influence the higher discriminability of the samples. Consequently, the overall results indicated that the different scale usage tendencies, as well as consensus procedures, influenced the sample configurations and discrimination, particularly with more distinct differences noted in the two consumer-oriented methodologies than in DA.

The RV coefficient results showed that the DA result had relatively low correlations with the FCP result (0.74) and the PSP result (0.65). The lower similarity between DA and PSP might be explained by the fact that PSP evaluated the samples based on holistic similarity, whereas the intensities of each attribute were measured in DA and FCP. However, the relatively low RV coefficient between DA and FCP (0.74) and the high RV coefficient between FCP and PSP (0.94) suggest that the overall RV coefficient results were influenced more by the inherent characteristics of consumers than by methodological differences. Only consensus terms were used in DA, whereas sensory attributes were generated individually in FCP. Similar to consumers in FCP, consumers in PSP expressed sensory attributes individually. These differences in generating discrepancy to some degree by resulting lower RV coefficients between DA and FCP, and DA and PSP. In a similar context, this discrepancy appears to be related to the heterogeneous and diverse characteristics of untrained consumers, who perceive and interpret sensory attributes differently from the trained panel in DA. Therefore, using DA could not guarantee the best results to discriminate subtly different samples. As it is difficult to clearly discern subtle differences across the samples even by trained panelists, approaching sample discrimination holistically would be a complementary method for the samples that have similar characteristics.

## 5. Conclusions

Overall, the mineral content of water samples was found to be the most influential factor affecting the sample discrimination of the three methodologies. In particular, the confidence ellipses of the results demonstrated that the samples were more distinguishable in the two consumer-oriented methodologies, whereas relatively few samples were discriminated in DA, showing that most samples had overlapping configurations. Despite the bland nature of the water samples, two consumer-based methodologies (FCP and PSP) provided reliable results, distinguishing more samples even with subtle differences in the RV coefficient. The present study confirmed that the utilization of these consumer profiling methodologies can be an effective alternative for investigating the sensory attributes of water samples. 

## Figures and Tables

**Table 1:** The water samples information and mineral compositions.

Sample Code	Ingredients	Manufacturing Location	Mineral Contents (mg/L) ^1^					Hardness (mg/L) ^2^
			Ca^2+^	Na^+^	K^+^	Mg^2+^	F^−^	
A1	Underground bedrock water	Jeju-si, Republic of Korea	3.5	6.1	2.7	2.6	0.0	19.8
A2	Underground bedrock water	Cheongdo-gun, Republic of Korea	21.1	14.7	0.7	5.8	0.3	77.1
A3	Underground bedrock water	Cheonan-si, Republic of Korea	49.5	11.2	1.7	10.6	0.3	167.5
B1	Deep sea drinking water	Sokcho-si, Republic of Korea	6.1	6.1	5.6	18.1	0.0	89.0
B2	Deep sea drinking water	Goseong-gun, Republic of Korea	5.3	7.4	5.4	14.9	0.0	74.2
C1	Purified water, calcium hydrogen carbonate	Jeju-si, Republic of Korea	4.4	5.6	37.6	3.0	0.0	22.3
D1	Underground bedrock water	Jilin, China	4.3	8.4	3.2	3.9	0.7	26.4
D2	Underground bedrock water	Auvergne, France	12.3	11.9	6.2	8.2	0.2	62.9
D3	Underground bedrock water	Viti Levu Yaqara, Fiji	17.6	15.2	4.3	12.9	0.3	98.4
D4	Underground glacial deposits water	Evian, France	76.8	6.7	1.0	24.7	0.0	295.0
E1	Filtered tap water	Jeonju-si, Republic of Korea	10.8	8.8	2.1	2.3	0.1	35.7

^1^ Mean values of duplicate measurements; ^2^ Hardness of water was calculated by the amount of Ca^2+^ × 2.5 + the amount of Mg^2+^ × 4.1.

**Table 2:** Sensory attributes generated from 11 water samples.

Descriptors	Definitions	Reference Materials	Intensity of Reference
Appearance			
Transparent	Degree of clearness	Tap water	15.0
Bubble	Amount of air bubbles in the inner surface of cup	Picture of sparkling water 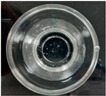	7.1
Taste/Flavor			
Sweet	Fundamental sweet taste in sucrose solution	0.5% (*w*/*w*) sucrose solution (CJ Cheiljedang Co., Seoul, Republic of Korea)	3.4
Sour	Fundamental sour taste in citric acid solution	0.01% (*w*/*w*) citric acid solution (Edentown F&B, Incheon, Republic of Korea)	3.3
Salty	Fundamental salt taste in NaCl solution	0.1% (*w*/*w*) NaCl solution (Bon salt; Hanju Co., Ulsan, Republic of Korea)	3.0
Bitter	Fundamental bitter taste in caffeine solution	0.02% (*w*/*w*) caffeine solution (Caffeine; Sigma-Aldrich, St. Louis, MO, USA)	4.7
Tap water flavor	Tap water flavor related to metallic and disinfectant flavor	20% (*w*/*w*) of tap water in filtered water	5.3
Mouthfeel			
Astringent	Tongue-shrinking, unclean feeling	0.02% (*w*/*w*) alum solution (Alum; McCormick & Co., Inc., Baltimore, MD, USA)	4.8
Cool	Cool and refreshing feeling	Filtered water (13 °C)	8.2
Heavy feeling	Smooth and heavy feeling related to mouth coating	0.1% (*w*/*w*) viscosity controlling agents (Visco-up, Rheosfood, Goyang-si, Republic of Korea; made by 500 rpm for 5 min)	6.6

**Table 3:** Mean intensity ratings of the sensory attributes for 11 water samples ^1,2^.

Samples	Appearance		Taste/Flavor					Mouthfeel		
	Clear	Bubble *** ^1^	Sweet	Sour	Salty ***	Bitter	Tap Water Flavor	Astringent	Cool	Heavy Feeling **
A1	15.0 ± 0.0	0.4 ± 0.3 ^b2^	1.5 ± 0.6	0.9 ± 0.5	0.7 ± 0.3 ^b^	1.8 ± 0.8	0.5 ± 0.4	1.7 ± 0.8	3.6 ± 1.3	1.0 ± 0.8 ^bc^
A2	15.0 ± 0.1	0.4 ± 0.3 ^b^	1.4 ± 0.5	0.9 ± 0.4	0.7 ± 0.4 ^b^	1.5 ± 0.7	0.5 ± 0.4	1.7 ± 0.7	3.4 ± 1.5	1.0 ± 0.6 ^abc^
A3	15.0 ± 0.1	0.4 ± 0.4 ^b^	1.3 ± 0.4	1.0 ± 0.4	0.9 ± 0.4 ^ab^	1.4 ± 0.5	0.6 ± 0.4	1.6 ± 0.7	3.1 ± 1.7	1.4 ± 0.9 ^ab^
B1	15.0 ± 0.1	1.6 ± 1.5 ^a^	1.5 ± 0.5	1.0 ± 0.5	0.8 ± 0.4 ^b^	1.5 ± 0.5	0.6 ± 0.5	1.7 ± 0.6	3.2 ± 1.6	1.2 ± 0.8 ^abc^
B2	15.0 ± 0.0	0.5 ± 0.4 ^b^	1.4 ± 0.5	0.9 ± 0.4	0.9 ± 0.5 ^b^	1.5 ± 0.6	0.7 ± 0.5	1.7 ± 0.7	3.1 ± 1.6	1.3 ± 0.7 ^abc^
C1	15.0 ± 0.0	0.5 ± 0.4 ^b^	1.4 ± 0.6	0.9 ± 0.5	0.8 ± 0.4 ^b^	1.7 ± 0.7	0.7 ± 0.6	1.7 ± 0.7	3.2 ± 1.5	1.2 ± 0.6 ^abc^
D1	15.0 ± 0.1	0.5 ± 0.4 ^b^	1.4 ± 0.6	0.9 ± 0.5	0.7 ± 0.4 ^b^	1.7 ± 0.7	0.6 ± 0.4	1.6 ± 0.7	3.4 ± 1.5	1.1 ± 0.6 ^abc^
D2	15.0 ± 0.0	0.4 ± 0.4 ^b^	1.4 ± 0.6	0.8 ± 0.4	0.8 ± 0.5 ^b^	1.4 ± 0.7	0.5 ± 0.4	1.7 ± 0.6	3.3 ± 1.5	1.2 ± 0.7 ^abc^
D3	15.0 ± 0.1	0.4 ± 0.4 ^b^	1.5 ± 0.6	0.8 ± 0.4	0.9 ± 0.4 ^ab^	1.5 ± 0.6	0.5 ± 0.4	1.6 ± 0.6	3.0 ± 1.4	1.4 ± 0.7 ^abc^
D4	15.0 ± 0.1	1.5 ± 1.5 ^a^	1.5 ± 0.6	1.0 ± 0.5	1.1 ± 0.5 ^a^	1.4 ± 0.5	0.5 ± 0.4	1.6 ± 0.6	2.9 ± 1.5	1.4 ± 0.8 ^a^
E1	15.0 ± 0.0	0.3 ± 0.3 ^b^	1.3 ± 0.6	1.0 ± 0.5	0.7 ± 0.3 ^b^	1.7 ± 0.7	0.5 ± 0.4	1.8 ± 0.7	3.3 ± 1.5	0.9 ± 0.4 ^c^

^1^ Significant differences at ** *p* < 0.01, *** *p* < 0.001. ^2^ Mean values with different alphabet stands for significant differences among samples.

**Table 4:** RV coefficients of descriptive analysis (DA), free-choice profiling (FCP), and polarized sensory positioning (PSP) results.

	DA	FCP	PSP
DA	1.00	0.74	0.65
FCP	0.74	1.00	0.94
PSP	0.65	0.94	1.00
MFA ^1^	0.82	0.98	0.96

^1^ MFA, multiple factor analysis.

## Data Availability

The data presented in this study are available on request from the corresponding author.
